# Barcoding T Cell Calcium Response Diversity with Methods for Automated and Accurate Analysis of Cell Signals (MAAACS)

**DOI:** 10.1371/journal.pcbi.1003245

**Published:** 2013-09-26

**Authors:** Audrey Salles, Cyrille Billaudeau, Arnauld Sergé, Anne-Marie Bernard, Marie-Claire Phélipot, Nicolas Bertaux, Mathieu Fallet, Pierre Grenot, Didier Marguet, Hai-Tao He, Yannick Hamon

**Affiliations:** 1 Centre d'Immunologie de Marseille-Luminy (CIML), Aix-Marseille University, UM2, Marseille, France; 2 Institut National de la Santé et de la Recherche Médicale (Inserm), U1104, Marseille, France; 3 Centre National de la Recherche Scientifique (CNRS) UMR7280, Marseille, France; 4 Institut Fresnel, Centre National de la Recherche Scientifique (CNRS) UMR7249, Marseille, France; 5 École Centrale Marseille, Technopôle de Château-Gombert, Marseille, France; Memorial Sloan-Kettering Cancer Center, United States of America

## Abstract

We introduce a series of experimental procedures enabling sensitive calcium monitoring in T cell populations by confocal video-microscopy. Tracking and post-acquisition analysis was performed using Methods for Automated and Accurate Analysis of Cell Signals (MAAACS), a fully customized program that associates a high throughput tracking algorithm, an intuitive reconnection routine and a statistical platform to provide, at a glance, the calcium barcode of a population of individual T-cells. Combined with a sensitive calcium probe, this method allowed us to unravel the heterogeneity in shape and intensity of the calcium response in T cell populations and especially in naive T cells, which display intracellular calcium oscillations upon stimulation by antigen presenting cells.

## Author Summary

The adaptive immune response to pathogen invasion requires the stimulation of lymphocytes by antigen-presenting cells. We hypothesized that investigating the dynamics of the T lymphocyte activation by monitoring intracellular calcium fluctuations might help explain the high specificity and selectivity of this phenomenon. However, the quantitative and exhaustive analysis of calcium fluctuations by video microscopy in the context of cell-to-cell contact is a tough challenge. To tackle this, we developed a complete solution named MAAACS (Methods for Automated and Accurate Analysis of Cell Signals), in order to automate the detection, cell tracking, raw data ordering and analysis of calcium signals. Our algorithm revealed that, when in contact with antigen-presenting cells, T lymphocytes generate oscillating calcium signals and not a massive and sustained calcium response as was originally thought. We anticipate our approach providing many more new insights into the molecular mechanisms triggering adaptive immunity.

## Introduction

Calcium ion acts as a universal second messenger in response to most cellular stimuli [1]. The pattern of the calcium response is biphasic, and primarily results from the production of inositol-3 phosphate (IP3) which triggers the release of calcium from the endoplasmic reticulum (ER store release) into the cytoplasm. This decrease is sensed by the stromal interaction molecules (STIM) that secondarily trigger the capacitative entry of extracellular calcium via the calcium release activated channels (CRAC) of the ORAI family [2]–[4]. Measuring the intracellular concentration of calcium is therefore of primary interest when analyzing transduction processes in living cells. Currently, this is achieved by methods which combine flow cytometry with intracellular diffusive fluorescent calcium-sensitive dyes in immunological relevant cells such as macrophages, NK cells, T or B cells. As an example, the calcium response is routinely monitored in T cells [5]–[15] as a functional read-out of the outside-in signal transduction subsequent to T-cell receptor (TCR) engagement by major histocompatibility complex (MHC) molecules with agonist peptide. However, when naive T cells encounter antigen-presenting cells (APC) and more generally when signaling is induced by intimate signaling-to-target cell-cell contact, flow cytometry approaches cannot fully recapitulate the physiological conditions of stimulation. In addition, recent works have demonstrated that TCR triggering by the MHC molecules follows unusual physico-kinetic parameters of serial engagement-disengagement [16], [17], which could be the molecular basis for the broad selectivity, high specificity, extreme sensitivity [18] and the capacity to induce a rapid intracellular response that characterize TCR triggering [19]. While soluble anti TCR or anti CD3 antibodies [20], antibody coated beads [21], [22], and phorbol myristate acetate/ionomycin [23] can all induce a productive calcium signal in T cells that ultimately leads to their activation, proliferation and cytokine production, the calcium elevation triggered by these strong irreversible stimuli is usually sustained. It may not therefore be representative of the response to physiological stimulations, which is more likely to consist in calcium spikes and oscillations [9], [24]–[26]. In order to capture the true calcium responses triggered during cell-cell contacts such as those occurring during T-cell and APC stimulation, video-imaging is compulsory in that it provides informative parameters on individual cell behavior (*i.e.* displacements, shape and intensity fluctuation) [27].

Obtaining such imaging data requires a complex custom-built experimental set-up usually dedicated to the detection of UV-excitable calcium probes and to the maintenance of physiological parameters for long-term recordings [9], [25], [28]. In any case cell tracking is mandatory and is often performed by manual approaches [28]–[30]; however, in addition to being time-consuming, manual analysis is prone to systematic errors due to subjective choice. Such pre-selection is an unavoidable step in any manual analysis. Automating the simultaneous tracking of hundreds of cells over hundreds of time frames would overcome these issues. Nevertheless, simultaneously tracking moving cells at high density represents a considerable challenge, particularly considering the need to correctly resolve interlaced tracks of stretching cells while providing valuable statistical confidence and robustness. While many software packages do incorporate a cell tracking module or plugins, the normalization of the calcium signal for each cell as well as the classification of calcium responses and any quantification generally have to be performed manually involving tedious excel datasheets [31].

Here we have developed a complete approach named Methods for Automated and Accurate Analysis of Cell Signals (MAAACS) which enables the simultaneous tracking of a population of individual moving cells (multiple target tracking, MTT) [32] and the automatic extraction of robust statistics on pertinent observables. The MAAACS program has been conditioned to synchronize, normalize and assemble the recorded cell traces to provide an at a glance calcium barcoding of a heterogeneous cell population and facilitate the *a posteriori* data mining and interpretation. We used MAAACS to examine the calcium responses induced in T cells upon interaction with APCs and with it were able to reveal the oscillatory calcium responses in mouse naive CD4+ T cells upon antigen recognition.

## Results

### Development of a protocol enabling the sensitive detection of intracellular calcium fluctuations in TCR mediated T-cell stimulation via its natural ligand

**Figure 1:**
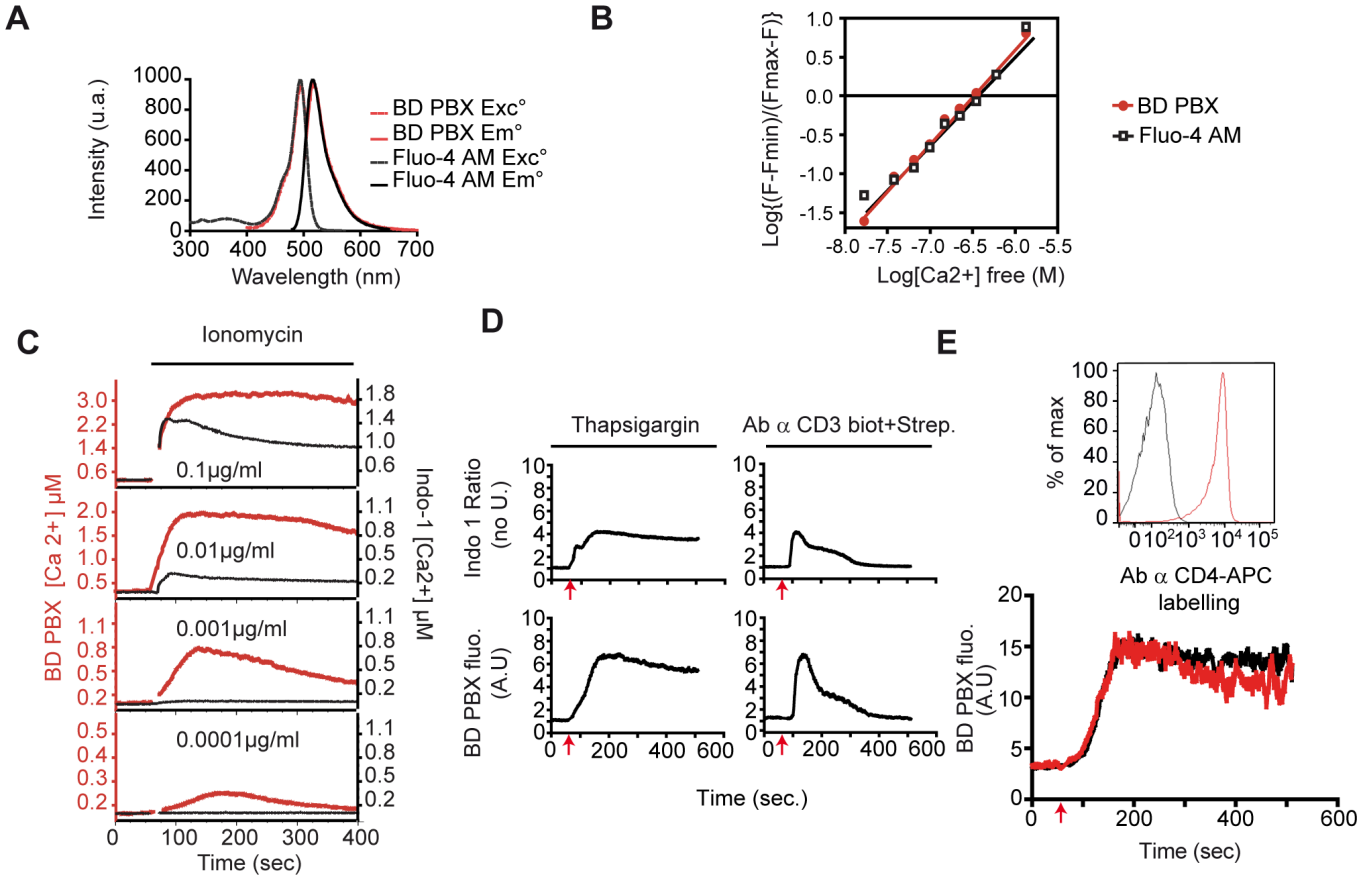
BD PBX, a highly sensitive calcium probe allows ratiometric analysis and antibody labeling of primary cells. (A) Comparison of excitation and emission spectra of Fluo-4-AM and the calcium indicator included in the PBX calcium assay kit, BD PBX. The excitation and emission intensity of Fluo-4 AM was exported from http://probes.invitrogen.com/media/spectra/data/14200ca.txt. To measure the spectrum of BD PBX, 3A9 hybridoma cells were loaded and analyzed with a spectrofluorimeter. Excitation intensity was measured for a fixed emission wavelength at 516 nm. Emission intensity was measured for a fixed excitation wavelength at 495 nm **(B) In vitro calibration of the BD PBX and Fluo-4 AM.** 3A9 T cells loaded with Fluo-4 AM or BD PBX as indicated in Materials and Methods, were washed three times in calcium free medium from the calcium calibration kit. Fluorescence of the cells was measured on a spectrofluorimeter from 500 nm to 650 nm. By a gradual increase of extracellular calcium concentration allow to plot extracellular calcium concentration as a function of the (F-F_min_)/(F_max_-F) ratio enabling to determine the Kd value. **(C). Comparison of calcium responses using Indo-1 or BD PBX upon various doses of calcium ionophore ionomycin.** 3A9 T cells were loaded with Indo-1 or BD PBX as indicated in Materials and Methods. Cells were stimulated 1 min after the start point with different concentration of ionomycin and fluorescence acquisitions were performed with a LSR I flow cytometer at 37°C. Fluorescence amplitudes or Indo-1 ratio were calibrated using the estimated in situ Kd of the Fluo-4 AM (1 µM) or Indo-1 AM (0.23 µM) with a scale factor of 2.9. **(D) Comparison of calcium responses using Indo-1 or BD PBX upon thapsigargin or anti-TCR crosslinking.** 3A9 T cells were loaded with Indo-1 or BD PBX as indicated in Materials and Methods. Cells were stimulated 1 min after the start point (red arrow) with thapsigargin, or a complex of antibody against CD3-biotin/streptavidin. Data were normalized to the baseline before stimulation to allow comparison between recordings. **(E) Calcium response upon thapsigargin stimulation of purified mouse CD4+ T lymphocytes.** Mouse CD4+ T lymphocytes were purified from spleen and lymph nodes and overnight serum starved. Cells were loaded with BD PBX and analyzed by flow cytometry under thapsigargin stimulation (red arrow in the right panel) with (red) or without (black) cell surface labeling with an anti CD4 coupled to APC antibody (left panel).

Aiming to establish an easy, robust, sensitive and reliable way of evaluating calcium fluctuations in T cells, we assessed many visible calcium indicators such as Fluo-4 AM, Fluo-3 AM, and Fluo-8. All displayed short term leakiness of the loading without membrane extruder blockers [33] (such as probenecid) [34] and subsequent intracellular compartmentalization, incompatible with unbiased calcium measurements [35]. In contrast, T-cell hybridomas (3A9) loaded with the calcium indicator BD PBX appeared to overcome most of these problems [36]. Compared to standard loading conditions, this procedure provided a stable loading of the fluorescent indicator (emission spectrum fully stackable with Fluo-4 AM; **Figure 1A**), without affecting cell viability (**Figure S1A**). We also documented that BD PBX fluorescence was photostable upon repetitive confocal illumination for 30 min, unlike Fura-red the fluorescence of which rapidly decreased (**Figure S1B**). This precluded us from performing BD PBX/Fura red ratiometric cytosolic calcium measurements [37], [38]. In addition, we investigated whether cytosolic calcium gradients could be visualized under such experimental conditions since few discrete hotspots were detectable among the homogenous fluorescence. BD PBX and mitotracker red loaded cells were imaged to decipher whether mitochondria would accumulate calcium indicator. In 3A9 T cells the two signals were not mutually exclusive (**Figure S1.C,D**) unlike in Jurkat cells or primary human T cells [35], [39]. Part of the signals was correlated under stimulation, most presumably caused by FRET between the two dyes (**Figure S1.C, D**). We detected no significant sequestration of the BD PBX calcium dye, unlike Fura-2 in Jurkat cells although this phenomenon had a limited impact on whole cell calcium measurement [35].

These inconsistencies with previous reports [34] motivated us to consider BD PBX as a close relative of Fluo-4 AM but harboring subtle differences, that required the dedicated loading buffer to avoid the dye leakage displayed by Fluo-4 AM (**Figure S1A**). Due to the lack of information about the BD PBX from the manufacturer, we thus determined the in vitro Kd of the BD PBX as described previously [40] (**Figure 1B**), which gave a consistent value for Kd of 312 nM±33, comparable to the Kd for Fluo-4 AM of 327±49 nM and that in the literature (Kd = 345 nM) [41] (see Materials and Methods). The great similarity between BD PBX and Fluo-4 AM led us to assume the in situ Kd of BD PBX to equal the reported in situ Kd value (1 µM) of Fluo-4 AM [34], deemed acceptable when accurate determination by electrophysiology is either not feasible or not available [28]. Based on this Kd value, we estimated that the intracellular calcium concentration in resting 3A9 T cell hybridomas would be around 90 nM and consistent with previously published values for these hybridomas [42] as well as leukemic cell lines Jurkat [39], mouse thymocytes [43] and human peripheral blood lymphocytes [35].

We used flow cytometry to compare the detection sensitivity of BD PBX with the ratiometric Indo-1 AM (routinely used in calcium assays, UV excitable). In terms of response pattern, T cell hybridomas loaded with BD PBX do not strictly speaking respond the same as those loaded with Indo-1 AM, as the non-ratiometric calcium indicator BD PBX does not allow the evaluation of intracellular free calcium concentration (**Figure 1C**). We therefore sought to estimate the intracellular calcium concentrations in BD PBX loaded T cell hybridomas. Upon various concentrations of ionomycin, we compared intracellular calcium elevation in BD PBX versus Indo-1 AM loaded 3A9 T cell hybridomas. As previously mentioned, the kinetics were not fully stackable and the calculated intracellular calcium concentration differed between the two methods due to Kd discrepancy and loss of linearity in the relationship linking calcium concentrations and high fluorescence amplitudes [40]. Indeed at such high fluorescence values under strong ionomycin concentrations, the calcium concentration was overestimated and is the reason for us reporting fluorescence amplitude instead of erroneous calcium concentrations throughout this manuscript. Nevertheless to our surprise, lower concentrations of ionomycin rapidly abrogated fluorescence elevations of indo-1 AM whereas BD PBX fluorescence elevation remained detectable even at subnanomolar ionomycin concentrations. This indicated that BD PBX is sensitive to low intracellular calcium elevation. Thapsigargin (**Figure 1D**) or the cross-linking of the TCR/CD3 complex by anti-CD3ε (2C11 biotin/streptavidin) induced in cells loaded with BD PBX responses similar to those induced by ionomycin. This method was extendable to naive primary CD4+ T cells labeled or not with an anti CD4 monoclonal antibody (**Figure 1E**).

### Automatic tracking of high density moving cells by MAAACS

**Figure 2:**
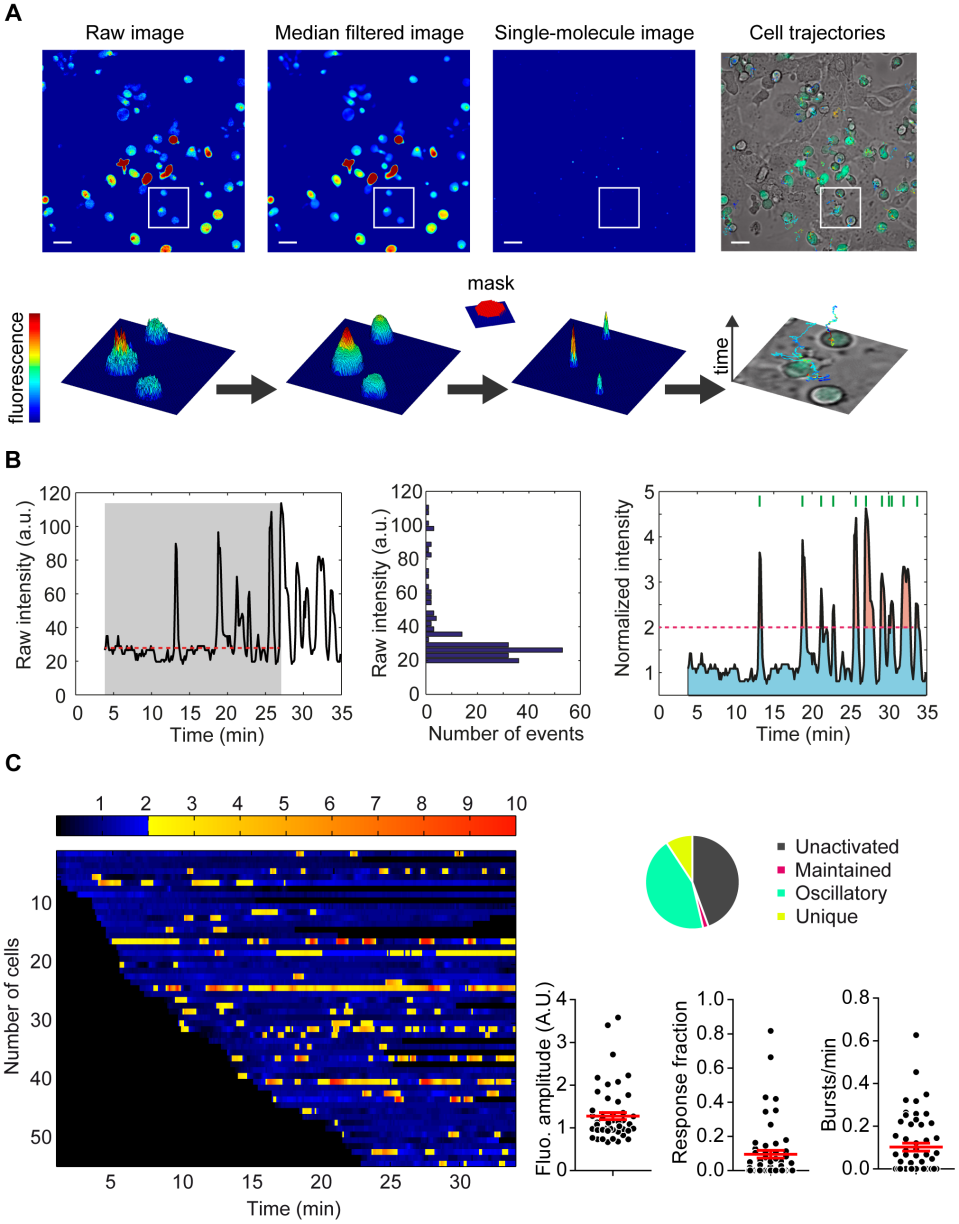
Calcium signal processing. (A) Automatic tracking of high density moving cells by MAAACS. Raw fluorescence experimental images (displayed with arbitrary pseudo-colors) are first filtered using a median filter to eliminate electronic noise emanating from PMT detection. The filtered image is correlated with a cylindrical mask (depicted next to the central arrow) to detect cells which are represented by Gaussian peaks keeping the overall initial intensities (height). Note that the width of the mask is set to adequately match the average cell width. Cell positions are finally linked from frame to frame following the MTT scheme to obtain trajectories that are color coded according to fluorescence intensity. Scale bar, 20 µm. The lower panel depicts the 2.5D views of the cropped areas. (**B**) **Automatic signal analysis.** Raw fluorescence intensities are normalized by taking into account the basal fluorescence enabling comparisons of the calcium signals between cells. The central panel represents the fluorescence intensity distribution before the maximal amplitude (gray area in the left panel). The median value (24.7 in this example, red dotted line in the left panel) is defined as the baseline for the cell. Raw intensities are thus normalized to this value (right panel). The calcium flux in each individual cell can be characterized by several analytical parameters concerned with its intensity (fluorescence amplitude and maximal amplitude) and its shape (response fraction, shown as a ratio of pink and blue areas, and number of bursts/min shown as green lines on the right panel). (**C**) **Graphical representations of the analytical parameters extracted from a movie treated with MAAACS.** The global response of cells is represented with a barcode view: the fluorescence amplitude of each cell is plotted along a horizontal line as a function of the time with a color coded intensity (dark to blue below the threshold of activation and yellow to red above the threshold of activation. Threshold of activation is set at 2 here). All analytical parameters (fluorescence amplitude, response fraction and number of bursts/min) extracted from the analysis with MAAACS are plotted in a scatter plot with the mean (+/−SEM, shown in red) and a pie-chart represents the cell response heterogeneity (inactive, maintained, oscillating and unique) (n_cells_ = 55).

While surface receptor crosslinking with antibodies is a convenient way of stimulating calcium responses in T cells, it cannot physiologically reproduce the dynamics of TCR/MHC-antigen interactions in the context of T-cell/APC contacts. Our goal was to decipher calcium signals arising from cellular contacts and requiring imaging approaches. We chose to perform all recordings on a conventional argon laser equipped-confocal scanning microscope, widely found in laboratories. Considering the lack of available methods [44] able to combine automatic tracking of cells and calcium signal processing, we set up our own procedure to automatically track moving cells at high densities with a minimum of input parameters. It is a customized version of our previously developed MTT algorithm [32], originally dedicated to tracking single fluorophores coupled to plasma membrane molecules at high density. We built a plugin that converts cellular images into cell position images that are comparable with the single molecule images supported by MTT (**Figure 2A** **& Figure S2**). Raw fluorescence images were first passed through a median filter to eliminate the electronic noise emanating from detection and an appropriate mask, consisting of a disk of adequate radius (see Materials and Methods for details on mask size), was applied to identify cells as single objects. Each cell was thus defined by the xy coordinates of its centroid which then served to reconstruct the cell's trajectory over the stack of images (**Figure 2A** **& Videos S1, S2, S3, S4, S5, S6, S7**). Noteworthy, more complex cellular shapes could be handled if using other appropriate detection schemes. Next, we generated synthetic images containing Gaussian peaks at the corresponding positions, with a radius optimized for the tracking performed by MTT and an intensity equal to the integrated cell signal, itself proportional to the intracellular calcium concentration. The resulting sequence of single molecule like images could then be treated by MTT (**Videos S5, S6**). Replacing each cell by a Gaussian peak of smaller size prevented the occurrence of two targets crossing over each other, initially a major concern for MTT, thus rendering the reconnection of traces during the MTT procedure far more efficient. Overlapping was then handled at the detection stage, where crossing cells presenting as a “peanut” shape were detected as two targets. However, strongly overlapping cells resulting in more of a spherical shape were detected as a single target and thus required z-stack acquisitions and appropriate analysis to recognize the occurrence of such crossing trajectories.

### Automatic signal analysis by MAAACS

MAAACS generates traces which are defined by the position of the detected cells over time and intrinsically calculates their instantaneous velocity and their fluorescence intensity. Given that the basal level varies from cell to cell due to differences in intracellular calcium concentrations, or due to heterogeneous efficiency in calcium indicator loading, we needed to accurately set a baseline of fluorescence that we defined as the median of fluorescence calculated until the maximum fluorescence value had been reached for each cell (**Figure 2B**). To establish the best mode of normalization, we analyzed for each cell in non-stimulating conditions how the mean signal amplitude was correlated with signal fluctuations. We found that this relationship was proportional, thus implying that the normalization can be performed by division. Calcium responses are highly diverse, both in terms of magnitude and oscillation (**Figure S3**). The amplitude of calcium mobilization varies according to the type of stimulus and the addition of inhibitors, but also within a cell population for a given stimulus/treatment. Moreover, the shape of these signals, their maintenance and their oscillations are also varied. We therefore defined analytical parameters to describe and characterize these response diversities (**Figure 2C**). For each cell, the response magnitude is described as the ***fluorescence amplitude (FA)*** of calcium mobilization, corresponding to the time-average of normalized intensities on the whole trace. The temporal fluctuations are deciphered by analyzing the persistence and the oscillations of the calcium signals. We defined as the ***response*** ** ***fraction (RF)*** the ratio of two phases: the time when the normalized intensity is greater than the threshold over the total time during which the intensity is detected. We also calculated the ***number of bursts/min*** **(BPM)** defining the number of peaks detected above the threshold divided by the duration of the detected trace (**Table S1**). In order to provide a global, comprehensive view of the calcium response in a substantial number of cells for any given condition, we color coded the normalized calcium intensities with a gradient of blue and orange for values below or above the threshold (see below), respectively. The resulting values, for each cell at each time-point, could then be pooled to generate a heat map, the dimensions of which hence corresponded to the cell number and time (**Figure 2C** **left panel**). Non-relevant pixels, either before or after detection of a given cell in the time-lapse movie, were left in black. This representation simultaneously depicts the global tendency, together with the intra-population variability of response [45]. Collectively, all calcium signal parameters are summarized on scatter dot plots where responding cells are represented by a single dot (**Figure 2C** **right panel**). By integrating these different parameters, the heterogeneous behavior of activated cells (maintained, oscillatory and unique) can be determined and inactive cells identified, the proportions of which are then represented in a pie chart diagram (see Materials and Methods for details on the classification).

### MAAACS tracking performance versus exhaustive manual tracking

**Figure 3:**
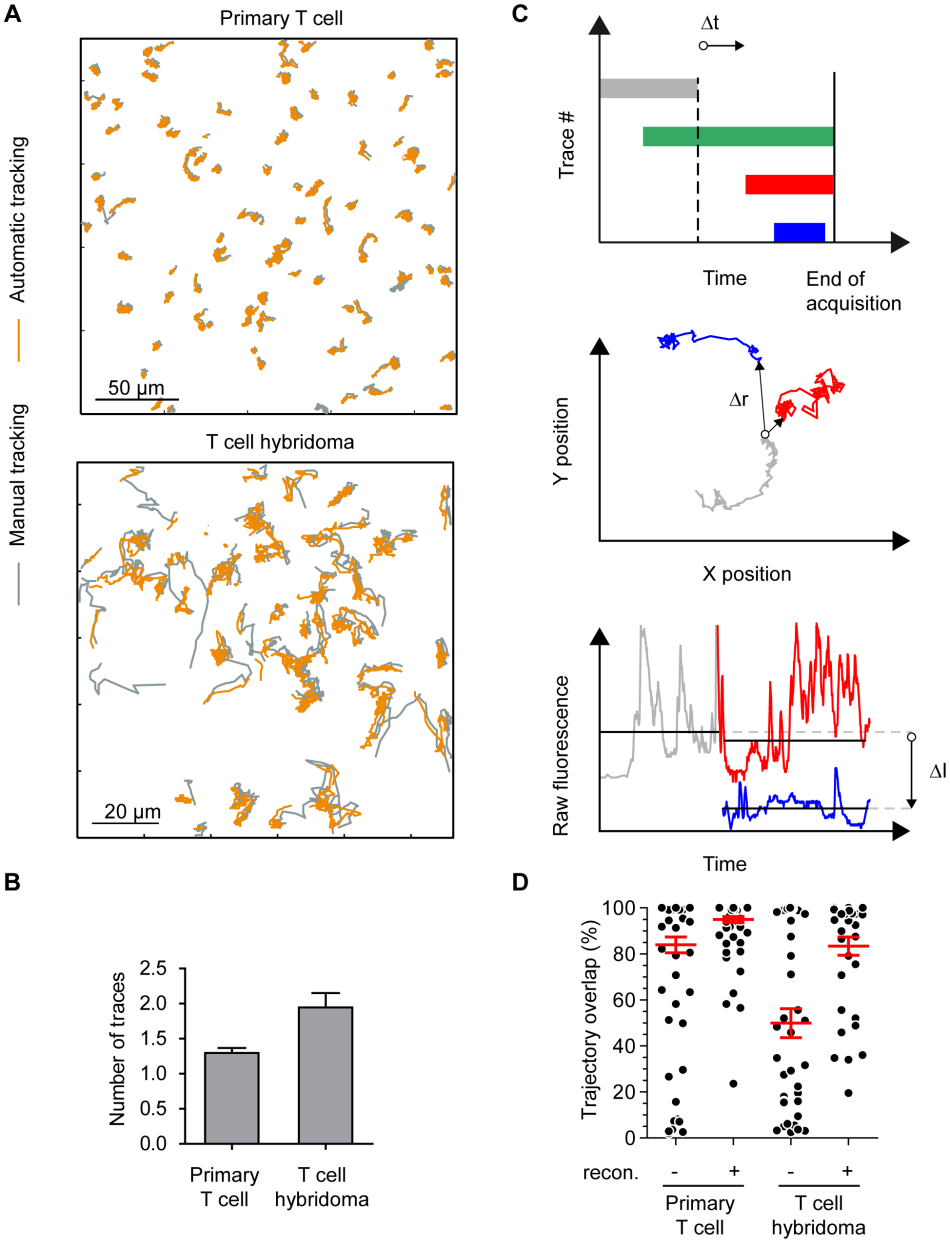
MAAACS tracking performance versus exhaustive manual tracking. (A) Overlay of cell trajectories analyzed manually or with MAAACS. Traces resulting from manual and automatic tracking (with MAAACS) are plotted together (respectively in orange and gray) to evaluate the tracking efficiency either for primary T cell or T cell hybridomas. **(B) Comparison of MAAACS tracking performances versus manual tracking.** Left panel - Each trajectory obtained with MAAACS is compared to the corresponding manual trace and the percentage of overlap is represented in a scatter plot for different cell types (primary T cells and T cell hybridomas) with or without reconnection (recon.). Right panel – For each MAAACS trajectory with reconnection, the average number of trace fragments are plotted (+/− SEM). **(C) Reconnection algorithm of MAAACS aborted traces.** An algorithm was implemented to reconnect trace fragments generated with the automatic tracking. Traces terminating before the end of acquisition (gray trace) were identified by the algorithm. Candidates for reconnection were selected among non-overlapping traces (blue and red traces, but not the green trace, starting before the end of the gray trace) following two additional criteria, narrow distance between traces and similar raw fluorescence amplitudes. The final decision is made using the Graphical User Interface (GUI). **(D) Impact of the reconnection on the trajectory overlap between manual tracking and MAAACS.** Scatter plot of the percentage of trajectory overlap with or without reconnection supervision calculated for each trace. The average (+/− SEM) are plotted in red.

An endemic problem in automatic tracking approaches is reaching a level of completeness that manual tracking only can guarantee. We analyzed several videos in parallel by the automatic and by manual methods and determined the percentage of cells tracked by MAAACS compared to that by manual tracking in the observation field (detection percentage). 3A9 T cell hybridomas or naive CD4+ T-cells loaded with BD PBX were seeded onto a monolayer of COS-7 cells stably expressing the molecules of the major histocompatibility complex [46]. We chose experimental situations with high cell densities on a rough and irregular surface. We only considered tracked cells detected by MAAACS over more than 5 images. The superimposition of the trajectories obtained manually or with MAAACS (**Figure 3A**) illustrated the efficiency of our algorithm, the detection percentage of which was greater than 96% (N = 125). Surprisingly, more traces were generated by MAAACS than obtained manually. Consequently, the MAAACS cell traces were fragmented into several parts as shown in **Figure 3B** illustrated by the number of fragments needed to reconstitute the full length trajectories (1.3 for primary T cell and 1.9 for hybridomas, **Figure 3B**). The lower efficiency for the latter can be explained by significant cellular shape changes over time preventing their detection by the circular mask and thus their reconnection. To fully document the cell response over time and to improve the quantitative analysis of cell signaling, we implemented a program allowing the reconnection of fragments of trajectories (**Figure 3C**). First, the method selected among the set of trajectories terminating before the end of the acquisition (plotted in gray in **Figure 3C**) to be reconnected to traces starting after the breaking off (plotted in red and blue in **Figure 3C**). Then the algorithm classified the candidates for reconnection by minimizing the interval between the stop and start times (Δt), the distance between the final and initial positions (Δr) and the difference of the mean fluorescence amplitude of the fragments (ΔI). The user is free to decide whether the trajectories should be reconnected by consulting the original video. In this way, the tracked time percentage was clearly improved (95% for primary T cells and 83% for hybridomas, **Figure 3D**).

### Threshold of specific activation

Within the course of this study, we noticed that without specific stimulation, cytoplasmic calcium concentrations displayed spontaneous oscillations, the amplitude of which was almost negligible for T cell hybridomas but not for primary naive T cells [13]. To account for this, we performed a median filtering with a sliding window of 7 frame-size on each fluorescence amplitude to remove aspecific oscillations in the absence of any stimulus. It was then important to carefully define the threshold of peptide-specific activation. As mentioned by many authors [26], [42], [47] and in our observations (**Figure 3**), calcium signaling exhibits high diversity even within the same cell line and depends on the applied perturbations (stimulation, drug treatment or mutation). It should be noted that defining any biological threshold for activation could be misleading since it is highly dependent upon the experimental conditions. Moreover, the definition of a criterion for specific activation should respect the response heterogeneity without favoring a subset of responding cells. Accordingly, we set up a detector which compares the fluorescence amplitude to a threshold of activation in order to identify activated cells in our experiments (**Figure S4**). If the fluorescence amplitude is greater than the threshold, then the cell is declared as activated. To determine this threshold, we investigated the statistical properties of the fluorescence amplitude of cells in the absence of any stimulation compared to that in cells that have been activated as a result of stimulation. For a given probability of false alarm (PFA), an activation threshold could be deduced from the cell responses in the absence of any stimulation. The probability of detection (PD) could then be calculated as the percentage of activated cells revealed by the detector. We then aimed to identify threshold values that minimized false detections (low PFA) without decreasing the identification of activating cell (high PD). A robust method to objectively determine the activation threshold is to perform a receiver operating characteristic analysis (ROC curves in **Figure S4A, B**) to explore the values of activation threshold that maximize the overall score PD x (1-PFA). All activation thresholds are reported in **Figure S4C**. Surprisingly, the activation threshold was quite stable whatever the cell type (hybridoma or naive T cells) or stimulation process (antibody or APC). For T-cell hybridomas, this method exhibited very high PD (>0.99) and low PFA (<0.02). PD was also high in primary T cells (>0.98) and the PFA was reasonable (<0.08) though slightly higher due to a higher diversity in the cell signaling.

### Evaluation of the calcium response of a heterogeneous population of individual T cell hybridomas

**Figure 4:**
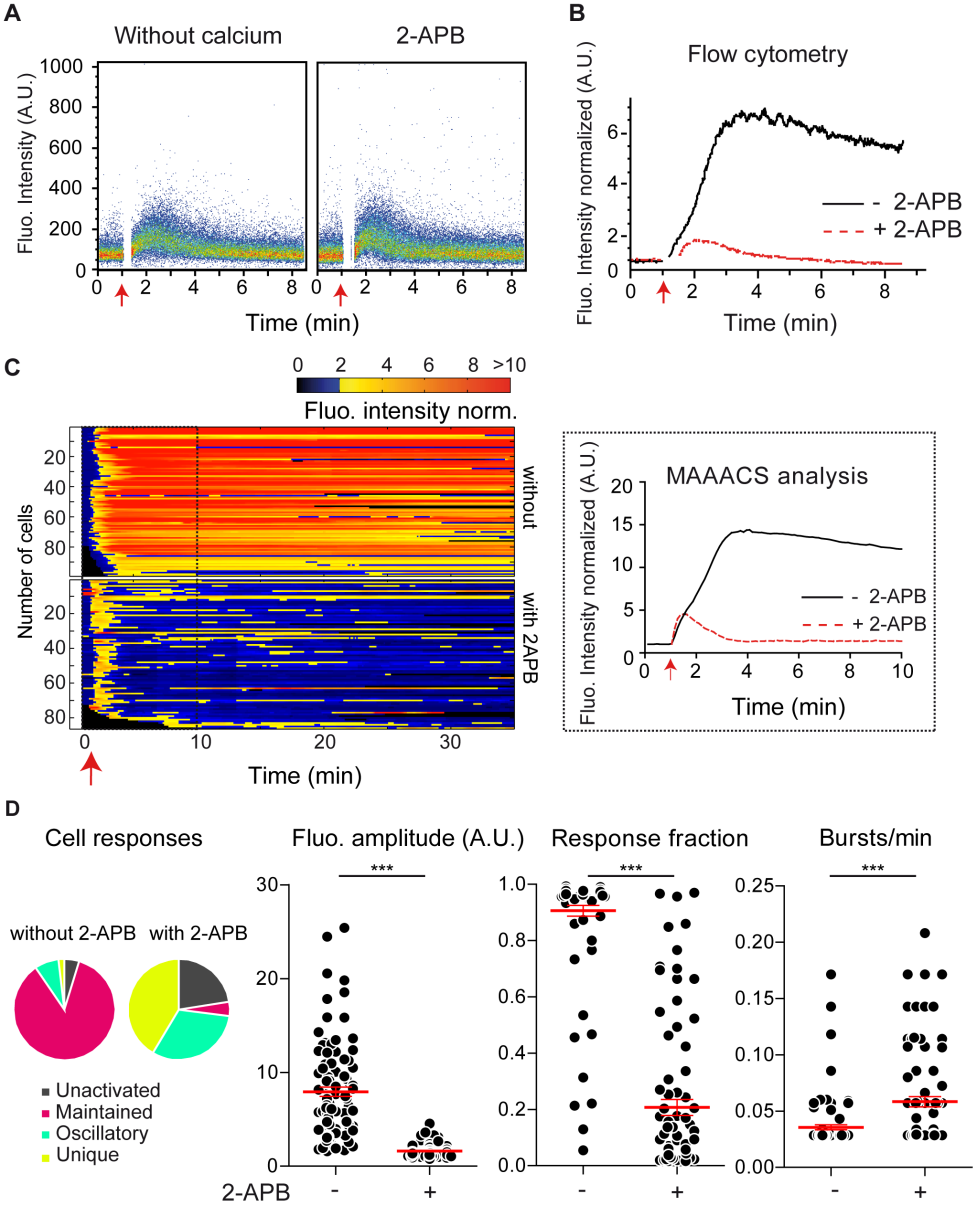
2-APB induced inhibition of CRAC channel activity prior to thapsigargin stimulation evaluated using flow cytometry as compared to confocal imaging analysis using MAAACS. (A) Intracellular calcium mobilization measured in HBSS without calcium and magnesium or by using the CRAC channel inhibitor 2-APB. 3A9 loaded T cells were resuspended in HBSS Hepes without Ca^2+^/Mg^2+^ or in HBSS Hepes with 2-APB before stimulation with thapsigargin (red arrow) and analyzed by flow cytometry. Cell fluorescence is represented on a pseudo-colored dot plot. **(B) Global and intracellular calcium mobilization evaluated by flow cytometry.** 3A9 loaded cells were stimulated with thapsigargin in the absence (−2-APB) or in the presence (+2-APB) of 2-APB. Evolution of the normalized median of fluorescence of the population is plotted vs. time. **(C) Global and intracellular calcium mobilization evaluated by confocal microscopy using MAAACS for detection and analysis.** 3A9 loaded cells were stimulated with thapsigargin (red arrow) in the absence (−2-APB) or in the presence (+2-APB) of 2-APB and fluorescence was monitored for 35 min under a confocal microscope. Videos were subjected to MAAACS analysis, and the average fluorescence of the tracked cells is plotted across time (left panel). For better comparison with flow cytometry, images from the first 10 minutes of recording are zoomed in (right panel). **(D) Analytical parameters of calcium signaling under thapsigargin stimulation in the absence or presence of 2-APB.** The fluorescence amplitude, the response fraction and the number of bursts/min are analyzed as a scatter plot (n_cells_ = 104, −2-APB; n_cells_ = 111, +2-APB). The mean value +/− SEM is represented in red in each condition. Statistical tests were carried out using the Mann Whitney non-parametric test (*** = p<0.001; ** = 0.001<p<0.01; * = 0.01<p<0.05; ns = p>0.).

**Figure 5:**
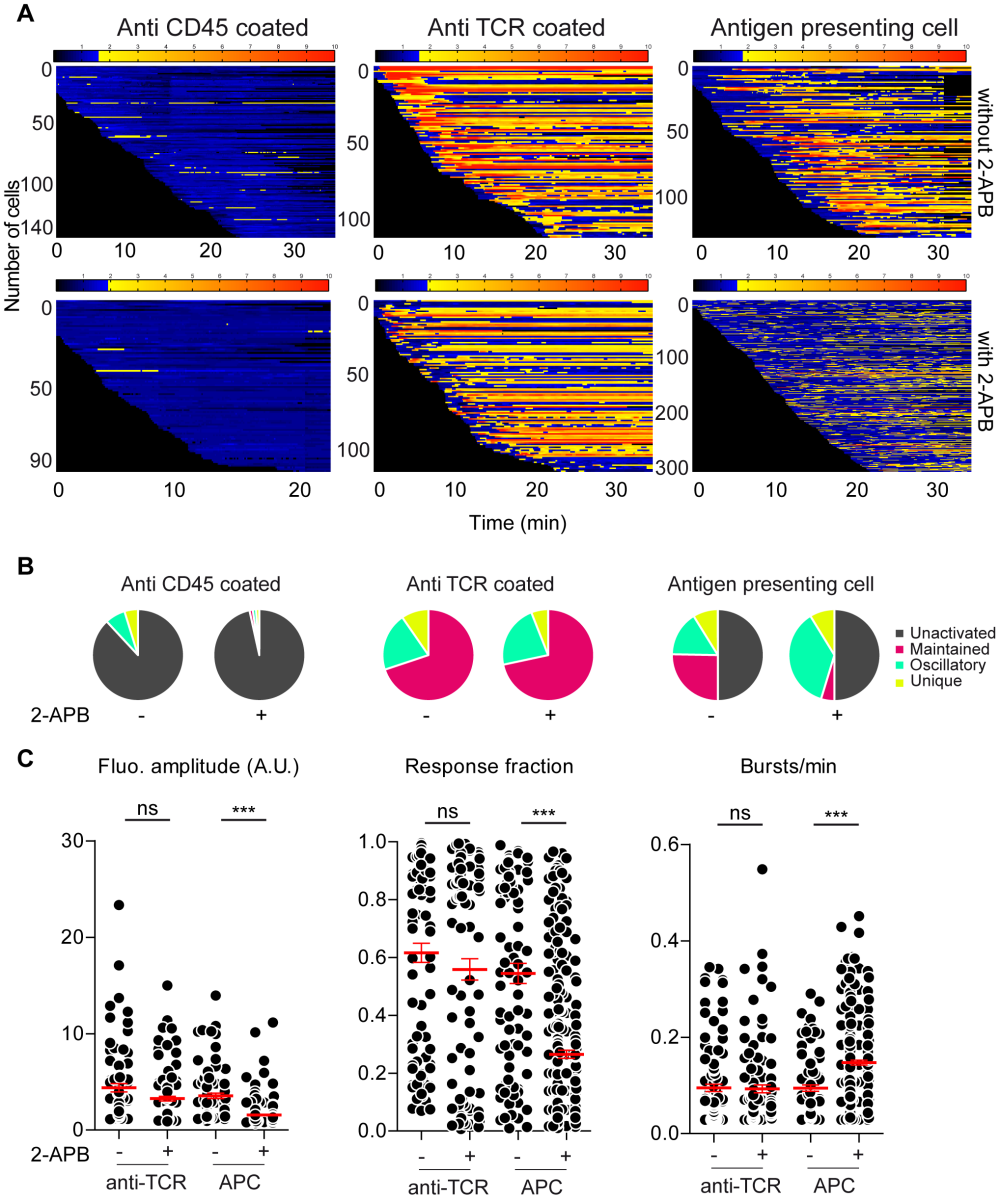
Evaluation of calcium response of a heterogeneous population of individual T cell hybridomas upon different experimental stimuli. (A) Barcoding of stimulation. The fluorescence intensity of all loaded and responsive (except for negative control conditions, i.e. anti CD45 coated surface) 3A9 T cell hybridomas acquired by confocal microscopy and detected by MAAACS are normalized and displayed as a barcoded response over time (color-coded as indicated). Anti CD45 antibody coated surface (left panel): Cells were seeded onto anti CD45 antibody coated Lab-tek chambers in the presence or absence of 2-APB. All tracked cells are represented on the barcode. Anti TCR antibody coated surface (middle panel): Cells were seeded onto anti TCR antibody coated Lab-tek chambers with or without 2-APB. Antigen presenting cell (right panel): Cells were seeded onto COS-7 experimental antigen presenting cells (see materials and methods) with or without 2-APB. (B) Mode of calcium response: To compare the calcium signal among different populations of individual cells with different types of stimuli we classified cells into four classes according to the intensity, number of bursts and response fraction. For each stimulus, these are represented as a pie chart: unactivated in gray, maintained in pink, oscillating in green and unique in yellow (C) Analytical parameters of calcium signaling for different stimuli (anti TCR antibody coated surface and antigen presenting cells): fluorescence amplitude, response fraction and bursts/min. The mean value (+/− SEM) of each analytical parameter is shown for each activated cell as a dot on each scatter plot. A non-parametric two-tailed unpaired Mann-Whitney test was used. *** indicates a p-value<0.0001. Activation thresholds (TH_act_) were 1.7 for hybridomas stimulated with antibody without 2-APB, 1.92 with 2-APB, 1.80 for APC without 2-APB and 1.62 without 2-APB) (n_cells_ = 146, anti CD45 − 2-APB; n_cells_ = 96, anti CD45 + 2-APB n_cells_ = 128, anti TCR − 2-APB; n_cells_ = 152, anti TCR + 2-APB; n_cells_ = 209, APC − 2-APB; n_cells_ = 515, APC + 2-APB).

To test our MAAACS algorithm, we analyzed a population of T hybridomas 3A9 loaded with BD PBX and seeded at the bottom of a well of a Lab-Tek before their stimulation with thapsigargin after 1 minute. We generated a sequence with a frequency of 1 confocal image every 7 seconds for 30 minutes. Fluorescence intensity was not affected by repetitive illuminations as previously mentioned (**Figure S1B**). No *a priori* assumption was made and raw recordings were subjected to MAAACS. To compare the calcium signals, we normalized the fluorescence intensities as a fold of the basal fluorescence. A mean curve of variations in fluorescence over time was obtained (**Figure 4C**) and compared to flow cytometry measurements (**Figure 4B**). Fluorescence rose immediately after thapsigargin addition, reaching a plateau 3 minutes after induction [48] before slowly decreasing (**Figure 4C**). This response was fully stackable with the kinetics monitored by flow cytometry (**Figure 4B**). We noted that the response/baseline ratio was higher according to imaging recordings, presumably due to a better sensitivity of detection on confocal photomultipliers. In this case the benefit of MAAACS is limited since all individual cells responded homogenously by a strong, sustained non-oscillating response (fluorescence amplitude, FA = 7.93±0.50; response fraction, RF = 0.91±0.02; bursts per minute, BPM = 0.04±0.001). In the presence of the CRAC channel blocker 2-aminoethoxydiphenyl borate (2-APB) [49], thapsigargin induced a weak elevation of fluorescence that was similar to Ca^2+^/Mg^2+^ deficient incubation conditions [48] (**Figure 4A**) and consistent between the two methods (**Figure 4B, C**). The analysis of the global tendency clearly masked the singularity of each individual cell. Through MAAACS analysis, while 50% of the cells displayed a unique rise of fluorescence as the average tendency would have suggested, the other half was equally composed of cells displaying sustained or oscillatory behavior. This implies that within a cell population and for any given stimulus, the observed differences in response are not limited to intensity dispersion but also to the mode of response. We sought to document this point using different experimental stimuli each producing their own calcium response in terms of shape, intensity and heterogeneity (**Figure 5**) [50]. Indeed, when the cells were seeded onto an activating surface (anti TCR antibody coated pits), while most responses were sustained (FA = 4.4±0.43, RF = 0.62±0.03, BPM = 0.09±0.01), heterogeneous responses were also observed. These heterogeneities were even more obvious when the 3A9 cells were stimulated by I-A^K-HEL^ expressing COS-7 APCs. The asynchronous landing of the T cells and the heterogeneous MHC II agonist peptide expression levels are parameters that affect the calcium response in addition to irregular crawling and scanning activities of the T cells on an APC monolayer. Indeed, during the first 30 minutes, 50% of the cells displayed a specific calcium rise [15]. Most of these exhibited a maintained fluorescence amplification but which was weaker in term of intensity and response fraction as compared to that in response to the stimulating antibody (**Figure 5C**), supporting the notion that abundant, immobile, highly affine ligands are not strictly recapitulating the stimulation by the natural membrane ligand of the TCR. Supporting this view, we analyzed the cell motility by MAAACS, as an integral parameter of T cell activation [29], [42], [51]. MAAACS analysis of cell velocity showed that inducers of strong and sustained calcium responses (thapsigargin or anti TCR coated slides and to a lesser extent anti CD45 unstimulating surfaces) negatively impacted the motility of cells [12], since instant speed measurements did not exceed 2 µm/min in the few minutes after landing on the slide, indicating that the cells were almost immobile. In contrast, T-cells migrating on COS-7 I-A^K-HEL^ displayed higher velocities (unactivated: 5.1±2.4 µm/min; activated: 3.7±1.2 µm/min) with a high mobile fraction (unactivated: 0.48±0.17; activated: 0.56±0.16). These velocities are fully consistent with 2-photon-microscopy measurements (**Figure S5**), where migratory T cells in lymph nodes, or thymus slices display mean velocities around 4 µm/min [9], [28], [52]–[54] or stimulating lipid bilayers [55].

Although experimental, COS-7 I-A^K-HEL^ have been shown to efficiently simulate physiological situations of T cell activation by inducing productive calcium signals, in a context of immunological kinapses [56], [57] rather than stable immunological synapses leading to specific cytokine secretion such as Interleukin-2 (data not shown). Additionally, as previously demonstrated, a clear correlation exists between activation and motility since unactivated T cells appear to move faster than activated ones within the same population [58] and T cell hybridoma mobility appears to decrease rapidly after calcium rise and rounding of the cells [10], [12] (**Figure S6A**). MAAACS was conditioned to automatically analyze the velocity and shape of the cells in addition to fluorescence signals, although no link between these parameters was found that was as tight as previous reports in cell systems expressing co-stimulatory or specific adhesion molecules.

In the presence of 2-APB, the fluorescence amplitudes upon either anti TCR (FA = 3.26±0.24) or COS-7 I-A^K-HEL^ stimulation (FA = 1.56±0.06) were strongly reduced compared to CRAC active control conditions. However, although the response fraction and the number of bursts per minute were left unaffected (RF = 0.56±0.03, BPM = 0.09±0.02) (**Figure 5C**) upon anti-TCR T cell activation, short and low calcium oscillations [26] were predominant in most T-cells (RF = 0.26±0.01, BPM = 0.14±0.01) (**Figure 5C** **and Figure S3B**) seeded onto COS-7 I-A^K-HEL^.

**Figure 6:**
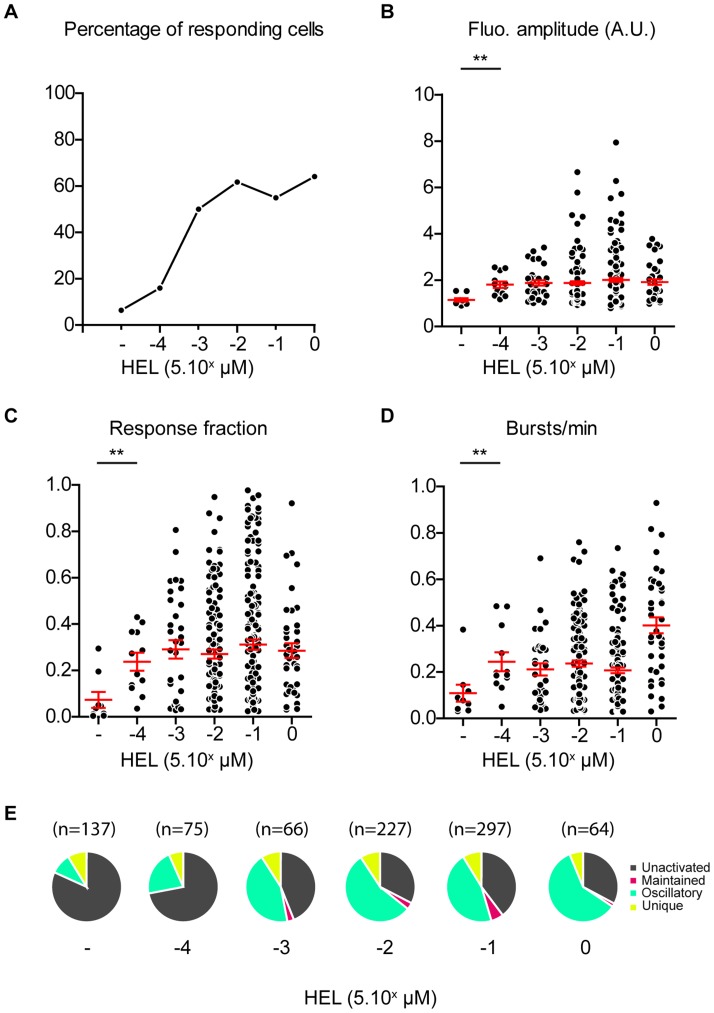
Effect of peptide concentration on the ER release of calcium. COS-7 cells expressing MHC class II molecules I-A^K^ were loaded overnight with increasing concentrations of HEL 46–61 peptides. Calcium responses in 3A9 hybridomas with 2-APB were analyzed with MAAACS. **(A) Percentage of responding cells for various peptide concentrations** was obtained with activation thresholds (TH_act_) respectively for hybridomas stimulated with increasing doses of 1.4 (0.5 nM), 1.8 (5 nM), 1.7 (50 nM), 1.6 (500 nM), 1.5 (5 µM) with 2-APB. **(B) Fluorescence amplitude of calcium response** of activated cells as a function of peptide concentration is represented on a scatter plot. **(C) Response fraction** of activated cells as a function of peptide concentration is represented on a scatter plot. **(D) Frequency calcium response bursts** of activated cells as a function of peptide concentration is represented on a scatter plot. The mean value +/− SEM is represented in red. Statistical tests were carried out using Mann Whitney's non-parametric test (*** = p<0.001; ** = 0.001<p<0.01; * = 0.01<p<0.05; ns = p>0.) **(E) Mode of calcium response**: For each antigenic peptide concentration, modes of calcium response are represented as a pie chart: unactivated in gray, maintained in pink, oscillating in green and unique in yellow.

In this case, the lack of co-stimulatory or specific adhesion molecules that usually sustain T cell/APC interactions and signaling [59] suggested that following TCR engagement by MHC-peptide, signaling events would occur through waves such as displayed by the calcium oscillations [60]. We therefore titrated the TCR-dependent calcium signaling in the presence of 2-APB in T-cells as a function of the amount of peptide loaded onto COS-7 I-A^K^. As shown in **Figure 6**, the peptide-specific, 2-APB sensitive calcium response was dependent upon the amount of peptide presented by COS-7 I-A^K^. In the absence of peptide, about 5% of the cells displayed a weak but significant calcium response above threshold. The percentage of responding cells increased proportionally to the peptide concentration to reach a plateau at a HEL 46–61 peptide concentration of 50 nM (**Figure 6A**), while being constantly oscillatory (**Figure 6E**). The fluorescence amplitude of the responding cells was significantly higher than that observed in the absence of antigenic peptide, even at low antigen doses (down to 0.5 nM). (**Figure 6B**) Surprisingly, the fluorescence amplitude, response fraction, and burst frequency were independent of the antigen concentration (except at higher antigen concentrations) (**Figure 6B–D**). These data show that, at least in 3A9 T cell hybridomas, the TCR-mediated antigen-dependent ER-store-operated calcium response is digitally triggered irrespective of the antigenic peptide concentration. This is consistent with results from an earlier study [42] focusing however on the global calcium responses in 3A9 T cells.

### Naive CD4+ T cells display heterogeneous intracellular calcium fluxes

**Figure 7:**
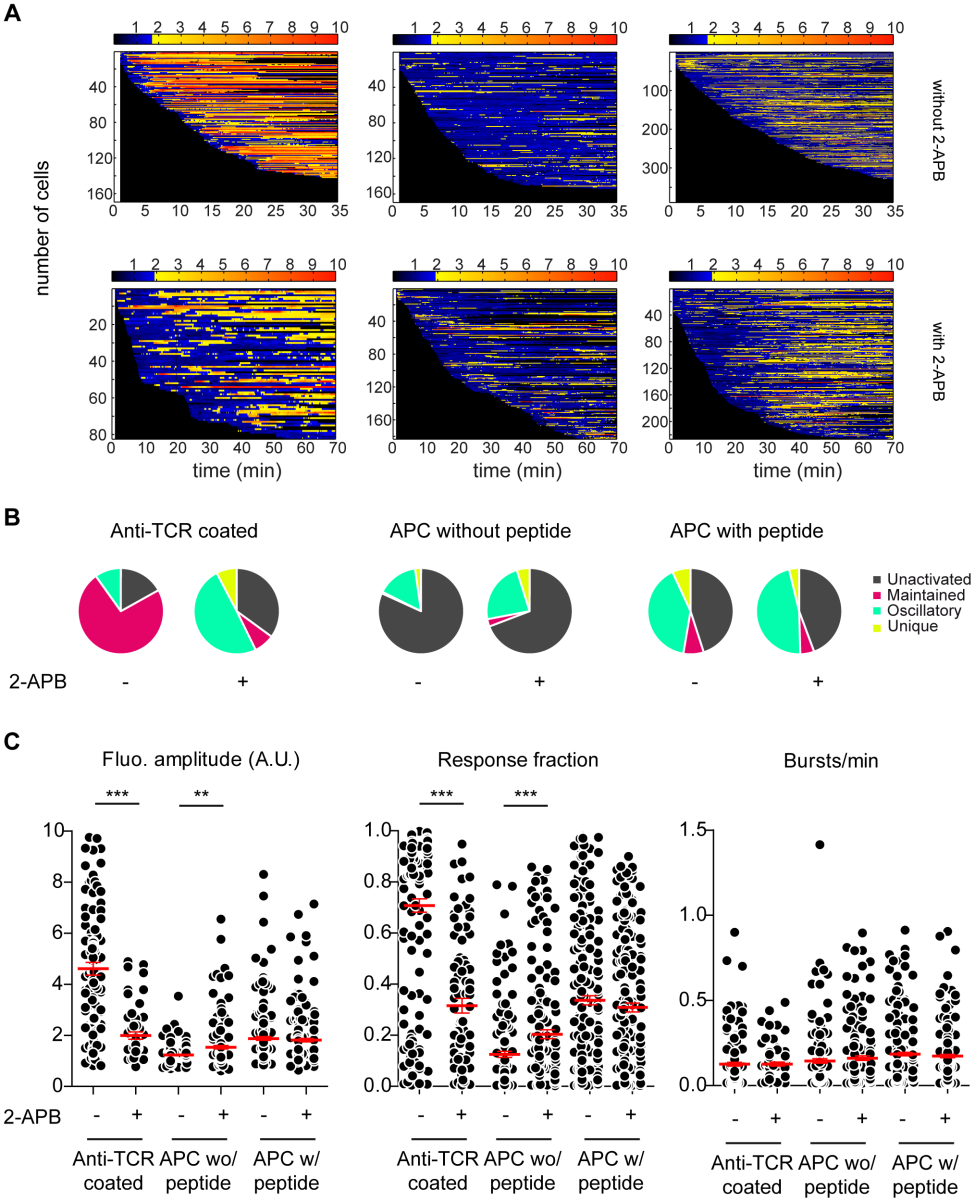
Naive CD4+ T cells display mainly intracellular calcium oscillations upon antigenic challenge. (A) Barcoding of stimulation. The fluorescence intensity of all loaded and responsive murine 3A9 naive CD4+ T cell acquired by confocal microscopy and detected by MAAACS are normalized and displayed as a barcoded response over time (color-coded as indicated). **Anti TCR antibody coated surface (left panel):** Cells were seeded onto anti TCR antibody coated Lab-tek chambers with (n_cells_ = 117) or without 2-APB (n_cells_ = 276). **Antigen presenting cell (middle and right panel):** Cells were seeded onto COS-7 experimental antigen presenting cells loaded or not with HEL peptides (see materials and methods) in the presence of 2-APB (n_cells_ = 266, without peptide; n_cells_ = 112, with peptide) or without 2-APB (n_cells_ = 491, without peptide; n_cells_ = 349 with peptide). **(B) Calcium response modes in naive CD4+ T cells** represented in pie charts (unactivated in gray, maintained in pink, oscillating in green and unique in yellow). **(C) Naive CD4+ T cell calcium responses upon different stimuli** revealed by MAAACS. Left panel - fluorescence amplitude of T cells in the absence or presence of 2-APB. Middle panel - The response fraction of T cells in the absence or presence of 2-APB. Right panel - frequency of calcium bursts in T cell in the absence or presence of 2-APB. Scatter plots only show signals from responding cells (TH_act_ = 1.74 without 2-APB, TH_act_ = 1.89 with 2-APB). The mean value +/− SEM is represented in red. Statistical tests were carried out with the Mann Whitney non-parametric test (*** = p<0.001; ** = 0.001<p<0.01; * = 0.01<p<0.05; ns = p>0.).

The antigen-dependent calcium response of mouse primary T cells has previously been investigated *in vivo* by microscopy on explants or sections of lymphoid organs [9], [28], [53] or *ex vivo* on artificial activating surfaces [55]. However, most of these studies were performed on lymphoblasts obtained by continuous activation in the presence of IL-2 for several hours and which therefore differ from naive T cells [18], [47], [61]. Data from studies that have examined the calcium response of naive T cells suggest that the calcium homeostasis of naive CD4+ T cells *ex vivo* is complex and at least in part antigen-independent [13], [62]. We evaluated these calcium responses of T-cells with MAAACS (**Figure 7**). Naive 3A9 transgenic CD4+ T cells [63], [64] seeded onto a surface coated with anti TCR antibodies showed a strong increase in fluorescence (FA = 4.61±0.25) that was maintained over time (RF = 0.71±0.03, BPM = 0.13±0.01) similar to that observed with 3A9 hybridomas (**Figure 7A, B** **,** **5C**). However, we also observed that when seeded onto non-stimulating I-A^K^ expressing COS-7 cells, around 20% of naive T cells responded spontaneously with short weak calcium pulses (FA = 1.23±0.03, RF = 0.13±0.01; BPM = 0.14±0.01) reminiscent of those previously reported [13] and [9]. Peptide specific calcium signals in the presence of COS-7 I-A^K-HEL^ were mostly oscillatory in more than 60% of the cells (FA = 2.02±0.09, RF = 0.32±0.02, BPM = 0.22±0.01), in contrast to those observed in hybridomas (**Figure 7B**) which were mainly sustained. Another fundamental difference with hybridomas is that we found no clear correlation between calcium fluxes and cell velocity (**Figure S6B**), which nevertheless was expected considering *in vivo* reports [65]. We then wondered whether these calcium responses in CD4+ naive T cells were sensitive to 2-APB. CRAC channel activity in T cells is characterized upon stimulation by thapsigargin or soluble anti-CD3 antibody (2C11), generating sustained calcium responses that are absent in patients suffering from an inherited form of severe combined immune deficiency (SCID) syndrome or upon 2-APB treatment [3] Indeed, upon 2-APB treatment, the thapsigargin-induced calcium response was drastically reduced. Equivalent kinetics (evaluated by flow cytometry) was obtained with 2C11 stimulation in the presence of 2-APB or EDTA (**Figure S7A–B**). No additive or competitive effect was detected under these experimental conditions (**Figure 7C**). In fact, 2-APB-treated naive T cells seeded onto anti TCR coated surfaces did show a calcium response (FA = 2.00±0.14, RF = 0.32±0.03, BPM = 0.13±0.01) that was greatly reduced compared to the native conditions without CRAC inhibitor (**Figure 7A**). More unexpectedly, 2-APB treatment did not induce a unique calcium peak suggesting that other calcium channels than SOCE mediate calcium entry in naive mouse T cells, since calcium oscillations are dependent upon calcium influx [66]–[68]. Similarly, we analyzed the calcium response to COS-7 I-A^K-HEL^ in the presence of 2-APB. There was a moderate but significant decrease in the amplitude of the calcium response (FA = 1.66±0.07, RF = 0.29±0.01, BPM = 0.16±0.01) compared to conditions in absence of 2-APB, together with a slight decrease of the oscillation frequency (**Figure 7A–C**). In addition 2-APB on naive T-cells seeded onto COS-7 I-A^K^ did not show any significant impact on calcium fluxes (FA = 1.28±0.05, RF = 0.13±0.02, BPM = 0.15±0.02), remaining lower to the calcium response in presence of antigenic peptides. Altogether upon blockade of the CRAC channel activity by 2-APB, we evidence that the SOCE dependent calcium entry plays a limited role in mouse naive T-cells upon TCR triggering by-MHC-peptide.

## Discussion

The first aim of this study was to significantly increase the sensitivity, accuracy, completeness and statistical reliability of video-microscopy approaches to record calcium fluxes, by combining a strong calcium probe with a robust algorithm for high density cell tracking coupled to an automated interface for rigorous post-acquisition analysis. The second objective was to use this method to describe the characteristic parameters of intracellular calcium fluxes within a population of T cells in response to different stimuli. We highlighted the heterogeneous nature and dynamics of these fluxes after TCR engagement by its natural ligand in a cell/cell context, which cannot be documented by flow cytometry. The TCR-MHC-peptide is a paradigm for unconventional intercellular receptor-ligand interaction [69] based upon successive cycles of engagement/release [16], [17]. However, more functional data supporting this current view are needed, taking care to account for the free motility of cells prospecting for cognate antigens supported by MHC molecules in a 2D cell membrane environment. Our goal was to develop experimental tools to contribute to the understanding of these mechanisms. The first challenge was finding a bright and stable fluorescent calcium probe in the visible range of the spectrum and that was easy to monitor both by cytometry and on conventional confocal microscopes.

T cells dedicated to calcium imaging are usually loaded with calcium indicators the emitted fluorescence of which is UV-shifted upon calcium elevation thus allowing ratiometric measurements (such as Fura-2). The sensitivity of these probes can however be impacted by their intracellular compartmentalization (adsorption by proteins, interaction with membranes or sequestration by organelles e.g. mitochondria), or their extrusion by organic cation transporters. To overcome these technical issues, loading can be performed with diluents (pluronic acid) or transporter blockers (probenecid), although these compounds can be noxious to T-cells and thereby affect their response [38]. Despite these potential drawbacks, Fura-2 loaded T cells are routinely used for long experimental procedure followed by transcriptomics without limitations by reduced cell viability [25].

In our study, cells loaded with BD PBX were used over extended periods of time without any evidence of mortality, compartmentalization, or photobleaching which have been reported to affect Fluo-4 AM use [34]. Although they could be considered as anecdotic or trivial, such properties enable more reproducibility and the use of BD PBX in a greater number of experiments compared to other fluorescent visible probes. Our here proposed MAAACS method incorporates our previously reported MTT algorithm dedicated to single particle tracking [32] and nanoscopy [70], [71] set up to enable the detection, monitoring and reconnection of trajectories of moving T cells acquired by conventional confocal microscopy. The ability to simultaneously track a great number of targets is in itself a challenge but in particular encounters difficulties when tracks are interlaced or crossing over. The performance of MTT was found to be slightly superior compared to existing algorithms however the implementation of a program of assistance proposing candidate traces to be reconnected to aborted traces was a major breakthrough in terms of improving the accuracy and completeness. In addition, during the analysis process, MAAACS enabled the rejection if necessary of dead or dividing cells. Consequently, while MAAACS is not yet a fully unsupervised method, we speculate that 3D time lapse video-acquisition methods (on a spinning disk confocal microscope, for example) would greatly reduce the number of aborted traces due to focus loss that occurs on a 2D+time acquisition scheme such as that in this study (in particular for primary T cells). Completeness is a major issue in this kind of study, since the baseline calculation could be under-estimated or incorrect when the first time points after cell landing are missing. Here, the automated normalization of calcium signals facilitates their comparison among a population of cells. The MAAACS analysis makes simple the analysis and, more importantly, the quantification of signaling. MAAACS deciphers a video sequence in about 10 minutes, where manual tracking and analysis would take at least 2 hours (depending on the duration of the time lapse and the number of cells). Video microscopy records the behavior of individual cells over time and not just part of a population of anonymous cells. This allowed us to demonstrate that calcium oscillations are highly diverse among cells [15] both in terms of intensity and frequency; they are mostly transient oscillations in primary T cells in contact with antigen loaded APCs. This diversity in cell responses supports the notion that T-cell triggering is stochastically linked to heterogeneity in the T cell population [50]. This is conceivable for T-cell clones due to genomic drift, but may seem more surprising for primary CD4+ T cells. Literature reports that oscillatory calcium fluctuations are associated to effector function of T cells and proliferation [21] in contrast to memory T cells which display unique increases in calcium [72]. In addition, sustained calcium responses are observed mainly in apoptotic T cells [73]. Altogether, our approach would be able to reveal in a seemingly homogenous population, T cell diversity in terms of function or fate, based upon antigen dependent calcium response mode. Another interesting finding is that CRAC channel dependent activity does not support a sustained calcium response in naive T cells encountering APCs, and that the predominant calcium response modes in T cells are oscillations, in agreement with literature [5], [9], [25], [74], at least in part sensitive to 2-APB blockade. This result supports recent works showing an intriguing role of voltage dependent Ca^2+^ channels (Ca_v_1.4) [66]–[68] in the calcium influx into naive T cells. Our results also suggest that membrane calcium channel openings are tightly correlated to ER-calcium waves upon TCR triggering [75], [76], and that sustained calcium fluxes such as those triggered by stimulating antibodies and revealed by flow cytometry or video imaging are not strictly physiologic, at least not in naive T-cells. It could be valuable to consider our results in the light of recent evidence suggesting a role for cytoplasmic calcium sustained elevation in the orientation of the cytoplasmic domains of the CD3 chains of the TCR/CD3 complex upon activation [77].

As a major conclusion, the introduction of MAAACS emphasizes the urgent need to record the effects of cell-to-cell stimuli using real-time videos. We believe that MAAACS holds huge scope that could be easily adapted to study various kinds of targets (such as Qdots, vesicles, cells, animals) based on various types of emitted signal, however one immediate application would be to compare our *in vitro* results to 2-photon imaging of calcium indicator-loaded T cells migrating in lymph nodes [53], [65].

## Materials and Methods

### Reagents and antibodies

2-Aminoethoxydiphenylborate (2-APB) (10 µM final concentration used for hybridomas, 20 µM for naive T cells) and thapsigargin (1 µM final concentration) were purchased from Calbiochem, and Ionomycin (0.1 µg/mL, final) from Sigma. The PBX calcium assay kit, the antibody against CD3ε (clone 145-2C11) (6.5 µg/ml, final), 2C11 biot (10 µg/mL final) and the F23.1 anti-TCR Vβ 8 1-3 antibody (10 µg/mL, final) were supplied by Becton Dickinson. The mitochondrion label, Mitotracker red CMX-Ros, and the calcium indicators Indo-1 AM, Fluo-4 AM and Fura Red AM were supplied by Life technologies (Molecular probes). Streptavidin (5 µg/mL) was supplied by Jackson Immunoresarch.C4H3 (anti I-Ak-HEL), GK1.5 (anti CD4), and H193.16.3 (anti CD45) (10 µg/mL, final) monoclonal antibodies were produced and purified in the lab from hybridoma supernatants according to standard protocols.

### Cell culture

3A9 hybridoma T CD4+ cells are specific for hen egg lysozyme peptide (HEL) bound to MHC II I-A^k^ molecules [78]. These cells were cultured in RPMI medium supplemented with 5% FCS, 1 mM sodium pyruvate and 10 mM Hepes.

COS-7 cells were cultured in DMEM medium with 5% FCS, 1 mM sodium pyruvate and 10 mM Hepes. Experimental antigen-presenting cells (APCs) were generated by stably transfecting COS-7 cells (Amaxa, V solution, A024) with plasmid cDNAs coding for the α chain of MHC II and the β chain of MHC II I-A^K^ alone or covalently fused to a peptide derived from HEL (provided by D.A. Vignali) [46]. Cells were sorted according to their positivity to surface labeling by C4H3 antibodies (Facsvantage, Becton Dickinson). APC monolayers were generated by seeding 5.5 10^4^ cells into poly-L-Lysine-coated 8-well Lab-Tek chamber (Nunc).

Spleens and lymph nodes were recovered from CBA/J x C3H non-transgenic mice and 3A9 TCR transgenic mice [63], [64]. After the extraction of cells onto nylon membrane in DMEM F12 medium (Lonza), splenic erythrocytes were removed via NH_4_Cl lysis. CD4+ T cells were isolated by depleting the CD4 negative cells according to manufacturer instructions (Dynal Mouse CD4 Negative Isolation Kit, Invitrogen).

### Calcium imaging

The day before experiments, cells were overnight serum starved in DMEM-F12 medium supplemented with 1% of Nutridoma-SP (Roche). For COS-7 transfected by plasmid cDNAs coding for the α and β chains of MHC II (I-A^K^), HEL peptide was added to the culture medium the day before the experiment. Coated surfaces were obtained by incubation with the appropriate concentration of antibody 24 h before the experiment at 37°C.

#### BD PBX

5 10^4^ T cells per well were plated in 96 well plates in 100 µL of complete medium. Cells were loaded with BD PBX diluted in 1X dye loading solution (according to manufacturer instructions) at 1/1000^e^ (∼1 µM) for 3A9 cells (1/1666 for primary T cells i.e. ∼0.6 µM). 100 µL of this solution were dispatched to each well before incubation at 37°C during 1 hour (30 min for primary T cells) in the dark. Cells were then washed twice in Hank's balanced salt solution (HBSS) Hepes buffer containing 1 mM calcium and resuspended in the same medium. Five wells were pooled (2.5 10^5^ cells) and analyzed either by flow cytometry or microscopy. When mentioned, cells were resuspended in HBSS without Ca^2+^/Mg^2+^, Hepes 1 mM. 2-APB was added just before recordings. Additional experiments were performed by adding Fura Red to the BD PBX dye loading buffer to a final concentration of 5.5 µM. Alternatively, Fluo-4 AM was added to the loading medium in the presence or in the absence of dye loading solution to a final concentration of 10 µM.

#### Indo-1 AM

2.5 10^6^ cells were resuspended in 500 µL of complete HBSS buffer (HBSS without calcium, without MgCl_2_, supplemented with 1 mM CaCl_2_, 1 mM MgCl_2_, 0.1% BSA and 1 mM Hepes) with 5 µL Indo-1 AM (10 µM) and incubated for 30 minutes at 37°C. Cells were washed twice in warm complete HBSS (37°C) and adjusted to the concentration of 5 10^5^ cells/mL in complete HBSS before acquisition.

### Flow cytometry

Cells were analyzed on a LSR I flow cytometer (Becton Dickinson) with Cell Quest software or LSR II (for Fura Red/BD PBX and Indo-1 AM acquisitions) using the FACSDiva software. PBX calcium indicator was observed over time on the FL1 channel with an excitation by an Argon laser 488 nm and a 530/30 nm emission filter at 37°C, maintained using a water bath. Data analysis was performed with FlowJo software and the median intensity of fluorescence was plotted vs. time after exclusion of dead cells and cell debris.

### Confocal microscopy

Movies were made on a Zeiss LSM 510 Meta confocal microscope equipped with a 30 mW argon laser (25% output, 1% AOTF). Pictures were taken with a C-Apochromat 40×/1.2 water immersion objective, using the 488 nm line of the argon laser, HFT UV/488 dichroic mirror and a 505 nm long pass filter at 37°C, maintained using a hot plate. Time-lapse movies were composed of 300 images (512×512 pixels; 8 bit; 225 µm×225 µm; pinhole set to 3 airy units) taken every 7 seconds. Additional observations were performed on an Ultraview VoX Perkin Elmer spinning disk confocal microscope.

### MAAACS analysis

All scripts, including multiple target tracking (MTT) [32], were developed under Matlab (The Mathworks). The source code of MTT, deposited at the *Agence pour la Protection des Programmes*, n° IDDN.FR.001.270021.000 S.P.2008.000.31230, is freely available for research purposes at http://www.ciml.univ-mrs.fr/lab/he-marguet.htm. Cell tracking and automated analysis of cell signals with MAAACS can be done either in command line (directly in Matlab) or using a graphical user interface (GUI). While the GUI is more intuitive it is limited to the analysis of a single acquisition whereas the command line solution permits the sequential analysis of several video-acquisitions.

#### Cell tracking with MAAACS

The exhaustive analysis of cell signals depends on an efficient tracking in the video acquisition stage, meaning an excellent detection and reconnection of cells from frame to frame. The MTT algorithm has already been optimized to efficiently track single molecule images. Thus, the conversion of cells into single molecules needs to be performed properly in order to allow the detection of all events in each frame. Mask optimization needs to be done for each and every cell type in order to mimic the cell shape and size. Too small a mask could produce multiple detections for the same cell, whereas a cell could be missed with too large a mask. The mask radius (in pixels) must therefore be set by taking into consideration the mean cell radius (R_cell_) and the pixel size on the image (L_pix_) at the magnification used during acquisition. The mask radius is then set to R_cell_/L_pix_.

#### Cell signal analysis with MAAACS

MAAACS automatically provides analytical parameters both on the fluorescence intensity and on the trajectory of cells. Mainly, it concerns their mean value, the persistence of high signal and the oscillation frequency. We have already developed the parameters for the fluorescence intensity (namely the fluorescence amplitude, the response fraction and the number of bursts/min). Similarly, this methodology has been used to analyze the cellular velocity: measured parameters were thus ***mean velocity*** (time-averaged speed) and the ***mobile fraction*** (ratio of two phases: the time when the speed is greater than the threshold over the total time the cell is detected).

In addition, we introduced an automatic method to classify the responses of activated cells. This enabled us to classify response as “maintained” when the response fraction was higher than 0.8 and “unique” when the response fraction was lower than 0.2 with a single burst. In other cases, calcium responses were defined as “oscillatory”. We considered the prototypic “maintained” response as the T-cell calcium response upon thapsigargin and the “unique and transient” response that upon thapsigargin with 2-APB. The distributions of these cell responses were finally plotted as a pie-chart, and revealed the behavior of a cell upon a given stimulation and treatment.

### Spectra analysis and *in vitro* Kd determination

Spectra of BD PBX and Fluo-4 AM were performed on the Cary Eclipse spectrofluorimeter (Varian). *In vitro* Kd determinations were performed using the calcium calibration kit (Life technologies) according to the manufacturer's instructions.

### Statistical analysis

All statistical analyses and normality tests were performed using GraphPad Prism 5.00. To determine the normality of the data, we performed a D'Agostino-Pearson normality test. Since not all our data were normally distributed, we used a non-parametric statistical test (two-tailed Mann-Whitney test with an alpha level of 5%).

## Supporting Information

Figure S1 **Evaluation of BD PBX as a reliable visible calcium indicator.** **(A) BD PBX fluorescence.** 3A9 T cells were loaded with Fluo-4 AM or BD PBX with or without PBX dye loading buffer. Cells were seeded onto poly-L Lysine coated labtek wells at 37°C and imaged under a spinning confocal microscope. The same wells were imaged after 30 min. **(B) BD PBX fluorescence unless Fura-red is stable under repetitive illuminations on a confocal microscope.** 3A9 T cells were loaded with BD PBX or Fura Red as indicated, and seeded onto poly-L Lysine coated labtek wells at 37°C and imaged under a confocal microscope. Arrival of all cells in the observation field was synchronized and the median fluorescence value was expressed as a function of time. Independent recordings were analyzed under similar experimental conditions. **(C) Evaluation of calcium gradient in BD PBX loaded T cell hybridomas.** 3A9 T cells were loaded with BD PBX and mitotracker red and imaged at 37°C on a spinning disk confocal microscope equipped with two simultaneous EMCCD cameras. Colocalisation plot were drawn. Cells were seeded onto non stimulating (NS) or stimulated (S) antibody coated labtek wells or onto COS-7 I-A^K^ APC loaded or not with HEL antigenic peptide. **(D) Snapshots of cells loaded concomitantly with BD PBX and mitotracker red in non-stimulating (NS) or stimulated (S) conditions.**
(TIF)

Figure S2 **Diagram of MAAACS methodology.** MAAACS methodology is mainly composed of three major data processing steps: The first step (blue panel) concerns the image filtering, the detection, the tracking of cells and the automated normalization of the fluorescence amplitude. The second one (optional, green panel) allows the definition of optimized activation thresholds by comparing experimental conditions (such as APCs with or without antigens). The last step (pink panel) automatically calculates a series of analytical parameters for each detected cell enabling the discrimination of activated and non-activated cells and their categorization based on mode of response.
(EPS)

Figure S3 **Diversity of calcium responses.** Single cell fluorescence recordings with MAAACS were performed on single 3A9 T cells stimulated by COS-7 I-A^K-HEL^ (**A**) without or (**B**) with the CRAC channel inhibitor 2-APB.
(EPS)

Figure S4 **Threshold calculation of calcium response.** The activation threshold is systematically evaluated both for 3A9 T cell hybridomas and naive T CD4+ cells and upon different way of stimuli (antibody or with antigen-presenting cell APC). The probabilities of false alarm (PFA) and the probability of detection (PD) are plotted as function of the activation threshold (named as ROC curves). They are respectively estimated considering the fluorescence amplitude of unactivated cells and activated cells upon each stimulation. Then the optimal activation threshold is calculated by maximizing the score PD x (1-PFA) (represented by a red dot). The determination of the threshold is done without 2-APB (**A**) and in presence of 2-APB (**B**). (**C**) Summary of the activation thresholds and their corresponding PFA and PD for 3A9 T cell hybridomas and naive T CD4+ cells, and upon different stimuli and treatments.
(EPS)

Figure S5 **Velocity of T cell hybridomas upon various stimuli.** **(A) Velocity Barcoding**: The velocity of each individual T cell (same cells (without 2-APB) as in **figure 5**) was concomitantly calculated with fluorescence intensity and displayed as a velocity barcode (color coded on a log scale). **Thapsigargin (TG):** Cells were stimulated with TG (n = 3 independent recordings). **Anti CD45 antibody coated surface:** Cells were seeded onto anti CD45 antibody coated Lab-tek chambers (n = 1). All tracked cells are represented on the barcode. **Anti TCR antibody coated surface:** Cells were seeded onto anti TCR antibody coated Lab-tek chambers (n = 2). **Antigen presenting cell (APC):** Cells were seeded onto COS-7 experimental antigen presenting cells with peptides (see materials and methods) (n = 8). (**B**) Superimposed trajectories of tracked 3A9 T cell hybridomas normalized to their starting coordinates (each cell trace has been plotted in a randomly chosen color). Upon stimulation with APC, the traces were plotted separately to distinguish the behavior of unactivated from activated cells. **(C) Analytical parameters for velocity under different stimuli**: The mean value (+/− SEM) for each analytical parameter (velocity, mobile fraction)) is shown in red for each activated cell depicted as a dot on each scatter plot.
(EPS)

Figure S6 **Diversity of correlation between cell velocities and calcium influx in T cell populations.** The velocity of a set of individual T cell was concomitantly color coded with fluorescence intensity and displayed as a function of time. (**A**) Panel of non-activated or activated 3A9 T cell hybridomas. (**B**) Panel of non-activated or activated primary naive CD4+ 3A9 T cells.
(EPS)

Figure S7 **CRAC channel activity in naive T cells.** **(A) Effect of EDTA on the calcium response within naive T CD4+ cells.** Naive CD4+ T cells were stimulated by anti CD3 antibody and calcium mobilization was measured by flow cytometry in the presence of increasing concentrations of EDTA. **(B) Global and intracellular mobilization of calcium in naive CD4+ T cells.** The effect of 2-APB on calcium mobilization was measured by flow cytometry in naive CD4+ T cells after stimulation by thapsigargin. **(C) Comparison of the effects of EDTA and 2-APB on intracellular calcium mobilization.** The effects of 2-APB and EDTA were compared by flow cytometry in naive CD4+ T cells after stimulation by anti CD3 antibody.
(EPS)

Table S1 **Summary of all analytical parameters introduced and used by MAAACS.**
(EPS)

Video S1 **Raw fluorescence movie of T cells loaded with calcium indicator (Methods).**
(MOV)

Video S2 **Raw fluorescence movie of T cells loaded with calcium indicator in a 3D representation of a zone of Video S1.**
(MOV)

Video S3 **Median filtered movie corresponding to Video S1.**
(MOV)

Video S4 **Median filtered movie corresponding to Video S1 in a 3D representation of a zone of video S3.**
(MOV)

Video S5 **Single-molecule like movie corresponding to Video S1.**
(MOV)

Video S6 **Single-molecule like movie corresponding to Video S1 in a 3D representation of a zone of Video S5.**
(MOV)

Video S7 **Trajectories of the cells from Video S1 over each frame as reconstructed by MAAACS.**
(MOV)
