# Supplementary figures and images for: Barcoding T Cell Calcium Response Diversity with Methods for Automated and Accurate Analysis of Cell Signals (MAAACS)
Source: PLoS Comput Biol. 2013 Sep 26;9(9):e1003245. doi: 10.1371/journal.pcbi.1003245 (PMC3784497; doi:10.1371/journal.pcbi.1003245)

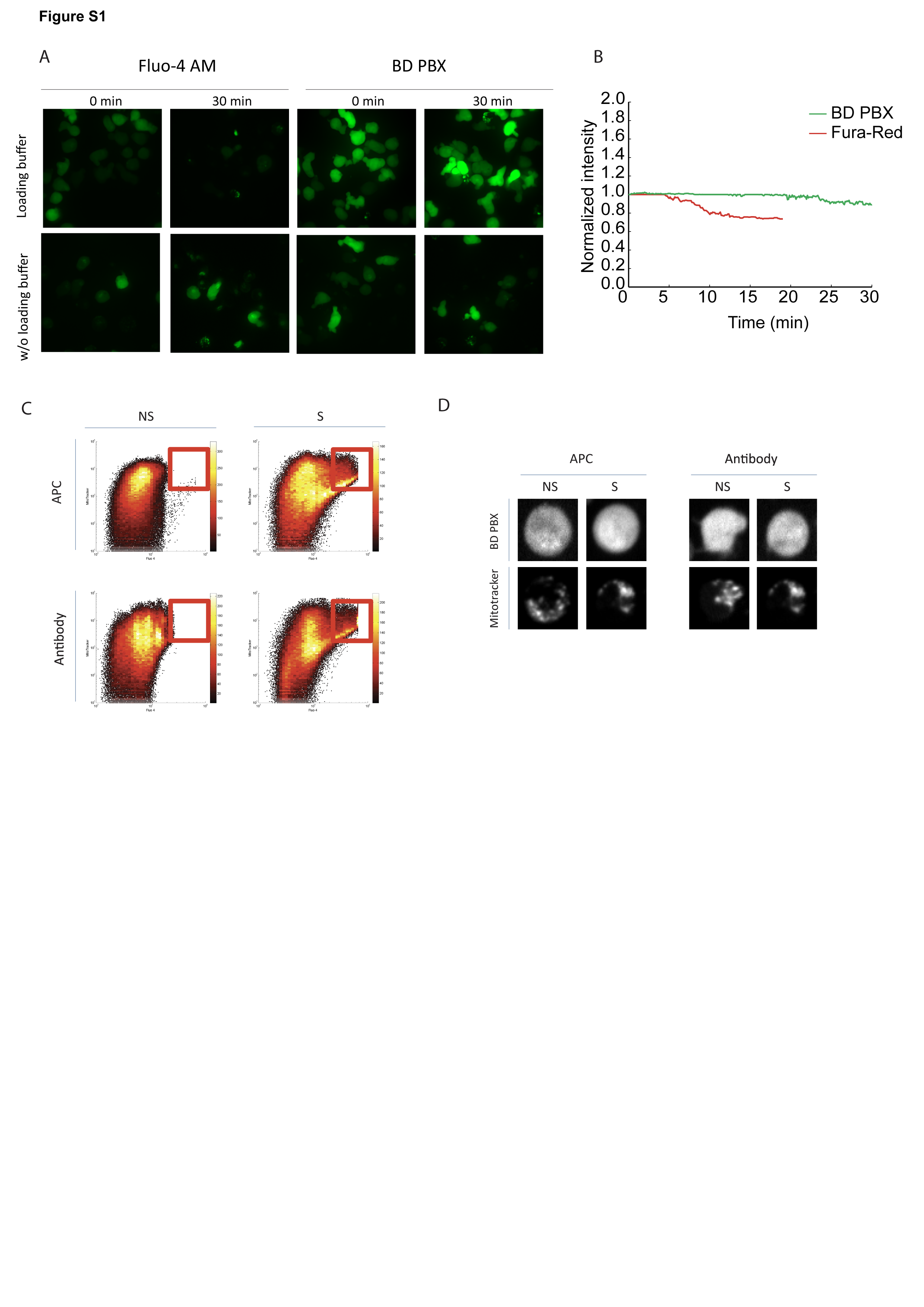

Supplement: Figure S1 — Evaluation of BD PBX as a reliable visible calcium indicator. (A) BD PBX fluorescence. 3A9 T cells were loaded with Fluo-4 AM or BD PBX with or without PBX dye loading buffer. Cells were seeded onto poly-L Lysine coated labtek wells at 37°C and imaged under a spinning confocal microscope. The same wells were imaged after 30 min. (B) BD PBX fluorescence unless Fura-red is stable under repetitive illuminations on a confocal microscope. 3A9 T cells were loaded with BD PBX or Fura Red as indicated, and seeded onto poly-L Lysine coated labtek wells at 37°C and imaged under a confocal microscope. Arrival of all cells in the observation field was synchronized and the median fluorescence value was expressed as a function of time. Independent recordings were analyzed under similar experimental conditions. (C) Evaluation of calcium gradient in BD PBX loaded T cell hybridomas. 3A9 T cells were loaded with BD PBX and mitotracker red and imaged at 37°C on a spinning disk confocal microscope equipped with two simultaneous EMCCD cameras. Colocalisation plot were drawn. Cells were seeded onto non stimulating (NS) or stimulated (S) antibody coated labtek wells or onto COS-7 I-AK APC loaded or not with HEL antigenic peptide. (D) Snapshots of cells loaded concomitantly with BD PBX and mitotracker red in non-stimulating (NS) or stimulated (S) conditions. (TIF) [file pcbi.1003245.s001.tif]
